# Neural Adaptations Associated with Interlimb Transfer in a Ballistic Wrist Flexion Task

**DOI:** 10.3389/fnhum.2016.00204

**Published:** 2016-05-03

**Authors:** Kathy L. Ruddy, Anne K. Rudolf, Barbara Kalkman, Maedbh King, Andreas Daffertshofer, Timothy J. Carroll, Richard G. Carson

**Affiliations:** ^1^Trinity College Institute of Neuroscience and School of Psychology, Trinity College DublinDublin, Ireland; ^2^School of Psychology, Queen’s University BelfastNorthern Ireland, UK; ^3^Neural Control of Movement Lab, ETH ZurichZurich, Switzerland; ^4^Department of Neurocognitive Psychology, Goethe UniversityFrankfurt, Germany; ^5^Faculty of Human Movement Sciences, Vrije University AmsterdamAmsterdam, Netherlands; ^6^Centre for Sensorimotor Performance, School of Human Movement Studies, University of QueenslandBrisbane, QLD, Australia

**Keywords:** cross education, contralateral adaptations, corticospinal, primary motor cortex, mirror training

## Abstract

Cross education is the process whereby training of one limb gives rise to increases in the subsequent performance of its opposite counterpart. The execution of many unilateral tasks is associated with increased excitability of corticospinal projections from primary motor cortex (M1) to the opposite limb. It has been proposed that these effects are causally related. Our aim was to establish whether changes in corticospinal excitability (CSE) arising from prior training of the opposite limb determine levels of interlimb transfer. We used three vision conditions shown previously to modulate the excitability of corticospinal projections to the inactive (right) limb during wrist flexion movements performed by the training (left) limb. These were: (1) mirrored visual feedback of the training limb; (2) no visual feedback of either limb; and (3) visual feedback of the inactive limb. Training comprised 300 discrete, ballistic wrist flexion movements executed as rapidly as possible. Performance of the right limb on the same task was assessed prior to, at the mid point of, and following left limb training. There was no evidence that variations in the excitability of corticospinal projections (assessed by transcranial magnetic stimulation (TMS)) to the inactive limb were associated with, or predictive of, the extent of interlimb transfer that was expressed. There were however associations between alterations in muscle activation dynamics observed for the untrained limb, and the degree of positive transfer that arose from training of the opposite limb. The results suggest that the acute adaptations that mediate the bilateral performance gains realized through unilateral practice of this ballistic wrist flexion task are mediated by neural elements other than those within M1 that are recruited at rest by single-pulse TMS.

## Introduction

Cross education is the phenomenon whereby training of one limb gives rise to increases in the subsequent performance of its opposite counterpart. The degree of cross education—i.e., the level of (positive) interlimb transfer, may be defined as the benefit derived by the untrained limb expressed as a proportion of the improvement in performance accrued by the trained limb. Despite interest in cross education having been sustained since its initial documentation by Scripture et al. (1894) a comprehensive explanation of the mediating neural mechanisms remains elusive. In view of the fact that the execution of many unilateral tasks is associated with increased excitability of corticospinal projections to the opposite limb (Hortobágyi et al., 2003; Carson et al., 2004) it has been proposed that interlimb transfer of training induced performance gains may be subserved by interactions between the primary motor cortices (e.g., Hinder et al., 2011). The more general conjecture is that bilateral cortical activity generated during unilateral training drives concurrent neural adaptations in both cerebral hemispheres (Hellebrandt, 1951).

In the context of ballistic tasks in which short-term unilateral practice of finger or thumb movements brings about bilateral increases in movement velocity or acceleration, rises in the excitability of corticospinal projections to the muscles of the untrained limb have been reported (Carroll et al., 2008; Lee et al., 2010; Hinder et al., 2011; Poh et al., 2013; Dickins et al., 2015; Reissig et al., 2015). Nonetheless, there is as yet no compelling evidence that any such changes are instrumentally related to the level of interlimb transfer (Ruddy and Carson, 2013). For example, increases in corticospinal excitability (CSE) are present for projections to homologs of muscles that do not make a *direct* mechanical contribution to the action that is trained (Carroll et al., 2008). Furthermore, with respect to ballistic movement tasks, no association has yet been established between variations in degree of cross education expressed across participants, and the extent to which elevations in the excitability of corticospinal projections to the muscles of the untrained limb are manifested (Carroll et al., 2008; Hinder et al., 2011; Dickins et al., 2015; Reissig et al., 2015).

We surmised that if it were possible to manipulate during the execution of unilateral ballistic training movements, a factor that modulates the excitability of descending projections to the homologous muscles of the opposite limb, we could assay consequential changes in the level of cross education. As we and others have demonstrated previously, in both rhythmic and discrete upper limb tasks, the excitability of corticospinal projections to the opposite (inactive) limb is modified by augmented (Carson et al., 2005) and mirrored (Garry et al., 2005; Carson and Ruddy, 2012) visual feedback of the moving limb. By extension, if such variations in CSE are instrumentally related to the processes that underlie cross education, unilateral training undertaken while attending to a mirror image of the moving limb should accentuate the degree of transfer to the untrained limb. Howatson et al. (2013) and Zult et al. (2014) have recently presented a similar line of reasoning.

In the context of tasks that demand maximal motor output, increases in the rate of force development are accompanied by elevation of EMG (electromyogram) amplitude throughout the initial (e.g., 100 ms) period of muscle activation (Van Cutsem et al., 1998; Aagaard et al., 2002). Previous reports suggest that the maximum firing rate of motor units recruited in ballistic contractions increases in response to chronic (e.g., 5 days per week for 12 weeks) training, and that there is an earlier recruitment of these motor units (Van Cutsem et al., 1998). As such adaptations represent a potent means through which the velocity/acceleration of *ballistic movements* can be increased (Barry et al., 2005) we reasoned that they might similarly mediate the gains in performance exhibited subsequently by an untrained limb.

The point has been made previously (e.g., Ruddy and Carson, 2013) that the permutation of neural adaptations mediating the interlimb transfer of performance gains—whether these are defined in terms of strength, skill, or other kinematic parameters, is likely to depend on the characteristics of the task. Relatedly, the absolute magnitude of a change in an outcome variable obtained for the untrained limb may depend upon the scope for gains in performance to be realized by the training limb. For example, the extent of the elevations in strength brought about by a resistance-training regime of 4 weeks duration may be quite distinct from the increases in acceleration induced by repetitions of a brisk movement over the course of 30 min. With a view to permitting comparison both between variants of a particular type of task, and across different tasks, the approach taken in the present investigation is to define degree of transfer as the magnitude of change observed for the untrained limb expressed relative to the magnitude of change observed for the trained limb. A key advantage of this approach is that the resulting measure of transfer gain is dimensionless, and thus amenable to comparison across a range of contexts. The scientific validity of this approach notwithstanding, it should be noted that there are instances in which the absolute magnitude of a gain in performance achieved for untrained limb is of practical significance (e.g., Dragert and Zehr, 2013; Magnus et al., 2013). We therefore take this opportunity to emphasise further, that the measure of interlimb transfer employed in the present study is relative rather than absolute.

The aims of the present study were threefold. The first aim was to examine the hypothesis that increases in performance arising from interlimb transfer are contingent upon elevations in CSE generated in the course of training movements performed by the opposite limb. In order to address this aim, we used three vision conditions shown previously to modulate the excitability of corticospinal projections to muscles of the inactive limb *during* wrist movements performed by the opposite limb. With respect to this aim, the null hypothesis is that manipulation of visual feedback does not influence the magnitude of interlimb transfer. The second aim was to determine whether there is a relationship between individual variations in CSE arising from training movements performed by the opposite limb, and the magnitude of interlimb transfer (i.e., the degree to which improvements in performance accrued by the training limb extend to, and are shared by, movements performed subsequently by the untrained limb). With respect to this aim, the null hypothesis is that variations in the levels of interlimb transfer are not associated with variations in CSE. We also sought to determine whether, for a ballistic movement task, the degree of positive interlimb transfer of performance is related to alterations in muscle activation dynamics. With respect to this final aim, the null hypothesis is that, while changes in muscle activation dynamics may be directly related to the absolute magnitude of the change in performance observed for the untrained limb, they are not directly related to the degree of transfer (i.e., the magnitude of change expressed relative to that observed for the trained limb).

## Materials and Methods

### Participants

Two experiments are reported. Thirty-six healthy volunteers (age 22.33 ± 3.64 (SD), 22 Female) participated in Experiment 1. Eighty-one healthy volunteers (age 22.79 ± 2.53 (SD), 39 Female) participated in Experiment 2. Motivated by the benefits of replication in decreasing the likelihood of false positive findings (Ioannidis, 2014) the latter experiment included the two visual feedback conditions from Experiment 1 for which the largest difference in behavioral outcomes was obtained. In addition, to increase statistical power with a view to undertaking tests of association (see below), and to further reduce the prospect of type 1 error (e.g., Button et al., 2013), the number of participants assigned to each condition was increased approximately threefold. All were right handed, and gave informed consent to procedures that were conducted in accordance with the Declaration of Helsinki, and approved by the relevant Queen’s University Belfast and Trinity College Dublin Ethics Committees.

### Apparatus and Procedures

The left limb executed the training movements. The performance of the right limb was tested prior to and following training. The participants were seated with forearms supported and stabilized in a neutral position with the elbows semi-flexed (100–120°). The angle between the upper arm and the torso was 15–20°.

The hands were secured at mid palm in manipulanda (instrumented to transduce angular displacement) mounted coaxially with the (flexion-extension) axes of rotation of the wrists. A contact switch was activated upon flexion of the wrist (from a neutral position), which was opposed by a stiffness load (≈ 0.67 Nm/θ—rad) i.e., the torque that resisted flexion of the wrist was proportional to the angular displacement of the wrist.

**Figure 1:**
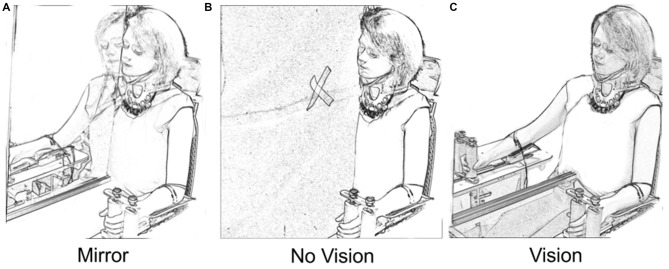
**Vision conditions.** In the “Mirror” condition **(A)** participants viewed the reflection of their moving left limb superimposed directly over the unseen position of the right limb. In the “No Vision” condition **(B)** the mirror was covered by a black drape. The participants were required to fixate upon a white cross that was placed on the drape in the line of sight between the eyes and the unseen position of the right limb. In the “Vision” condition **(C)** the participants viewed their inactive right limb. The position of the head was the same in the three vision conditions.

A mirror (depth 50 cm × height 90 cm) was aligned with the participant’s sagittal plane (Figure 1). As the mirror was incompletely silvered, it could first be positioned to ensure that the reflection of the left limb was superimposed precisely upon the directly sighted position of the right limb. When an opaque drape was placed behind the mirror, the partial transparency was eliminated (Mirror condition). The mirror could also be withdrawn, affording direct vision of the right arm (Vision condition). Placing the drape in front of the mirror eliminated both the reflected image of the left limb and direct vision of the right limb (No Vision condition). A white cross on the drape served as a point of fixation for participants in the latter condition. An orthopedic neck brace stabilized the head at ≈15° relative to the sagittal plane during behavioral testing/training, but not during assessments of CSE. This ensured that the angle of the head that was optimal for viewing of the reflected image in the Mirror condition was also adopted in the Vision and No Vision conditions.

### Electromyography

The electromyographic (EMG) activity of flexor carpi radialis (FCR) and extensor carpi radialis longus (ECR) was recorded from both arms using bipolar surface electrodes. EMG signals were amplified and bandpass (Experiment 1: 30 Hz–1 kHz; Experiment 2: 100 Hz–1 kHz) filtered. These and the transducer-derived voltages corresponding to displacements of the wrists were digitized at 2000 Hz during behavioral testing/training, and at 5000 Hz during the recording of motor evoked potentials (MEPs). Further details in relation to EMG signal processing and the adoption of filter settings are provided in the sections that follow.

### Transcranial Magnetic Stimulation (TMS)

A Magstim Rapid^2^ (Experiment 1) or Magstim 200 (Experiment 2) stimulator delivered magnetic stimuli via a (55 mm mid-diameter) figure of eight coil. This was placed over the primary motor cortex (M1) at the optimal position (“hot spot”) to obtain a motor evoked potential (MEP) in the FCR muscle of the contralateral arm. The coil was placed so that the axis of intersection between the two loops was oriented at approximately 45 degrees to the sagittal plane, such that the initial phase of the stimulating pulse induced posterior to anterior current flow across the motor strip. Once the hot spot was established, the lowest stimulation intensity at which MEPs with peak-to-peak amplitude of approximately 50 μV were evoked in at least 5 of 10 consecutive trials was taken as resting motor threshold (RMT).

MEP recruitment curves were obtained by delivering TMS at 10% increments of intensity between 90% and 150% of the RMT. Six stimuli were delivered at each level of intensity, except at 120% of RMT, in which case 12 pulses were delivered. The order of delivery was randomized. The interval between successive stimuli was 6 s. The total duration of the sequence was 4 min and 48 s. Each recruitment curve was used subsequently to derive a compound measure of CSE (Carson et al., 2013).

### Experimental Protocol

**Figure 2:**
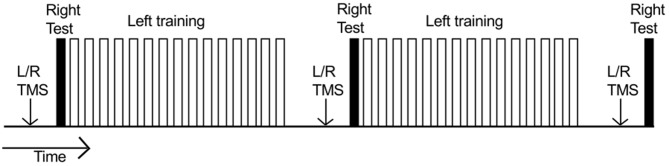
**Time course of an experiment.** Each unfilled bar represents a “trial” consisting of 10 training movements undertaken by the left limb. Two blocks of 15 trials were performed (300 training movements in total). Within each block, successive trials were separated by 30 s intervals. The right limb (filled bars) executed three separate series of 10 movements: (1) prior to left limb training; (2) 10 min after completion of the first block of training movements; and (3) 10 min after completion of the second block of training movements. Separate MEP recruitment curves (indicated by “L/R TMS”) were obtained: (1) prior to behavioral testing/training; (2) when 5 min had elapsed following completion of the first block of training movements; and (3) when 5 min had elapsed following completion of the second block of training movements.

During the course of training, each participant was required to undertake three hundred “fast as possible” discrete flexion movements of the left wrist. Each such ballistic movement was cued by the presentation of a tone (400 Hz sine wave). It was made clear to the participants that this was not a reaction time task, and there was no requirement to respond imperatively following the tone. Rather they could initiate the movement in their own time. In the course of a “trial”, 10 such movements were performed. Successive movements were separated by ≈ 7000 ms intervals. There were 15 trials in each of two “blocks” (total 300). Within each block, successive trials were separated by 30 s intervals (Figure 2). Five practice movements were first undertaken to familiarize the participant with the procedure.

Throughout training, the participants were encouraged to continually increase the peak acceleration of their wrist flexion movements. Feedback of performance was provided immediately following each movement (with the exception of the first two) by means of two qualitatively distinct auditory “sound bites”. One indicated that the peak acceleration of the movement was greater than the mean of the two preceding attempts. The other indicated that the mean peak acceleration of the preceding two attempts had not been surpassed.

Prior to the commencement of left limb training, each participant performed 10 “fast as possible” discrete flexion movements of the right wrist. No feedback of performance was provided following these movements. A further series of 10 right limb movements were performed 10 min after completion of the first block of (150) training movements undertaken by the left limb; and 10 min after completion of the second block of (150) training movements undertaken by the left limb (Figure 2).

Separate MEP recruitment curves were obtained for the right and left FCR prior to behavioral testing/training. Two further recruitment curves were obtained when 5 min had elapsed following completion of the first block of (150) training movements undertaken by the left limb (i.e., prior to the second series of 10 right limb movements). A third set of recruitment curves was obtained when 5 min had elapsed following completion of the second block of (150) training movements undertaken by the left limb (i.e., prior to the third series of 10 right limb movements; Figure 2). The order of the recruitment curves (left/right) was counterbalanced across participants.

### Manipulation of Visual Feedback

In Experiment 1, 12 participants were allocated in a quasi-random sequence to each of three visual feedback conditions. Those in the Mirror condition were asked to complete the training movements while looking at and attending to the reflected image of their left limb. In the Vision condition, participants were instructed to look at and attend to their quiescent right limb, while executing the training movements with their left limb. In the No Vision condition, participants were required to look at and attend to the white fixation cross on the drape covering the mirror, while executing the training movements with their left limb. In all instances in which the performance of the right limb was assessed, the No Vision condition was employed—whereby the participants were required to look at and attend to the white fixation cross.

In Experiment 2, 33 individuals were allocated to the Mirror condition. Due to the availability of additional recordings from a parallel study in which diffusion imaging (to perform white matter tractography) was undertaken on a separate occasion (10 participants), a total of 48 individuals were included in the No Vision condition. In all respects the procedures applied to the additional ten individuals were equivalent to those for others in the No Vision condition. It was simply the case that these participants attended an imaging suite on a separate occasion.

### Data Processing and Analysis

#### Kinematic Data

Following digital filtering (second order, dual pass Butterworth, low pass 6 Hz), the transducer derived displacement signals were differentiated twice to derive acceleration. The peak acceleration in wrist flexion was obtained for each movement. The mean peak acceleration of the 10 movements performed in each trial was then calculated.

The change in performance of the training (left) limb was calculated as: the mean peak acceleration of the trial wherein they performed best, minus the mean peak acceleration of the first 10 movements (i.e., trial 1), expressed as a percentage of the mean peak acceleration of trial 1. The change in performance of the untrained (right) limb was calculated as the mean peak acceleration of the 10 movements executed following the completion of left limb training (i.e., the Post trial), minus the mean peak acceleration of the 10 movements executed prior to the commencement of left limb training (i.e., the Pre trial), expressed as a percentage of the mean peak acceleration of the Pre trial.

Outliers were removed using the adjusted (for skewed distributions) boxplot method of Hubert and Vandervieren (2008). Data obtained from participants exhibiting a change in performance value for either the training or untrained limb that fell below the respective lower whiskers (in this instance set to the first quartile minus the interquartile range) were removed from all subsequent analyses. The magnitude of interlimb transfer was calculated for the remaining participants as the change in performance of the untrained (right) limb, expressed as a percentage of the change in performance of the training limb. In order to establish whether it could be inferred with sufficient confidence that interlimb transfer had occurred, bootstrapped (95%) confidence intervals were calculated (10,000 iterations) for the median transfer values obtained in each experimental condition. The rationale is that for instances in which the confidence interval does not enclose the value zero, it can reasonably be concluded that the effect was present.

#### Motor Evoked Potentials

In assessing CSE, the root mean square (rms) of the background EMG recorded in FCR and ECR was calculated for a window 100–3 ms before TMS onset (i.e., prior to the stimulus artifact). If the value was greater than 5 μV for either muscle, the corresponding MEP was disregarded. Overall, 97% of the recordings were retained. For those remaining, the mean (peak-to-peak) amplitude of the MEPs elicited at the seven respective stimulation intensities was calculated. For muscles proximal to the hand, the parameters derived from fitting procedures applied to MEP recruitment curves exhibit poor test-retest reliability (Malcolm et al., 2006; Carson et al., 2013). Indeed more generally, the assumptions underlying standard regression models are violated when used to fit MEP recruitment curves (Goetz et al., 2014). In order to overcome these limitations therefore, for each time of measurement (Pre, Mid and Post training), the summated area under the recruitment curve (AURC)—bounded by magnetic stimulation intensity and MEP amplitude (in units of mVT), was obtained using the trapezoidal rule. It has been demonstrated elsewhere (Carson et al., 2013) that the AURC is a very reliable measure of the state of corticospinal projections to hand and forearm muscles, which has construct, face, and concurrent validity.

#### Electromyographic Data (Experiment 2)

The EMG recordings obtained during the ballistic movements were high pass filtered digitally (second order, dual pass Butterworth) using a cutoff of 100 Hz. It has been shown previously (Potvin and Brown, 2004) that the portion of the EMG frequency spectrum thus removed does not materially affect estimates of the relationship between the EMG-envelope and force, although necessarily the selection of filter will determine the precise characteristics of the envelope.

The onset of activity in the FCR muscle preceding the onset of movement was obtained using a variant of approximated generalized likelihood-ratio (AGLR) test based detection described by Staude and Wolf (1999). The offset of activity was determined using an extension of the method outlined in Carson et al. (1995). The analytic envelope of the FCR EMG was first extracted using the Hilbert transform (Myers et al., 2003). This provides an estimate of the time varying amplitude of the compound motor action potential. The derivative of the enveloped signal was generated using a Savitzky-Golay polynomial, for which the window length of 157 ms corresponded approximately to a low pass filter cutoff of 10 Hz. The minimum value of the derivative in the period from the onset of the FCR EMG burst to the termination of the wrist flexion phase of the movement defined the FCR burst offset. The degree of co-contraction of ECR—a functional antagonist muscle in the context of wrist flexion, was quantified by calculating the root mean squared (RMS) activity of this muscle in the period between the onset and offset of the FCR burst. This value was expressed as a ratio of the FCR RMS activity recorded during the same interval.

**Figure 3:**
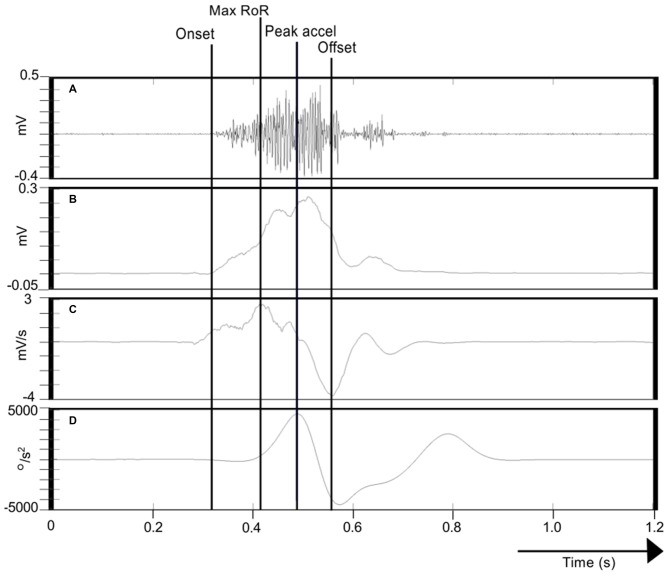
**Calculation of FCR EMG indices. (A)** Illustrates the Flexor carpi radialis/Electromyography (FCR EMG) trace recorded during one movement of the right limb. **(B)** Shows the corresponding Hilbert-transformed signal (analytic envelope), and **(C)** Its Savitzky-Golay first derivative. **(D)** Represents the acceleration trace associated with the movement. The onset of FCR EMG (cursor labeled “Onset”) was detected using a variant of the AGLR procedure. The peak acceleration of movement is also indicated (cursor “Peak accel”). The peak of the Savitzky-Golay derivative in the period from EMG onset to peak acceleration is marked as the maximum rate-of-rise (RoR) of the enveloped EMG time series (cursor “Peak accel”). The effective offset of FCR EMG activity (cursor “Offset”) was derived as the minimum of the Savitzky-Golay derivative in the period from the onset of the FCR EMG burst to the termination of the wrist flexion phase of the movement.

With a view to characterizing the activation dynamics of FCR—a primary agonist muscle in the present task, we calculated the maximum rate-of-rise (RoR) of the enveloped EMG time series (i.e., the maximum of the derivative) in the period from the burst onset to the time of maximum (wrist flexion) acceleration. The interval between the onset of FCR EMG activity and the time at which this event occurred was also recorded (Figure 3).

In addition to describing muscle activation profiles associated with the (voluntary) wrist flexion movements of the training limb, we also sought to determine whether activity could be detected simultaneously in the FCR and ECR muscles of the opposite (quiescent) limb. In the first instance, the burst detection algorithms used to detect the onset of activity in the left FCR were applied to the right FCR EMG recordings. The number of movements wherein an EMG burst was detected in right FCR was expressed as a percentage of the total number of training movements. As a means of characterizing activity in right FCR that was phase-locked to that of left FCR during generation of the training movements, the relative Hilbert phases (Gabor, 1946) between the derivatives of the respective enveloped EMG signals were computed within the periods of the left FCR bursts (e.g., Ridderikhoff et al., 2005). The dispersions of the phase angles (uniformity) were calculated following Mardia (2014). Uniformity takes values in the range 0 to 1, and is the directional equivalent of the inverse of the ordinary sample standard deviation on the line. All inferential analyses were conducted using the transformed uniformity measure (Mardia, 2014) thus permitting the use of tests based on standard normal theory.

## Results

### Experiment 1

#### Behavioral Outcomes

**Figure 4:**
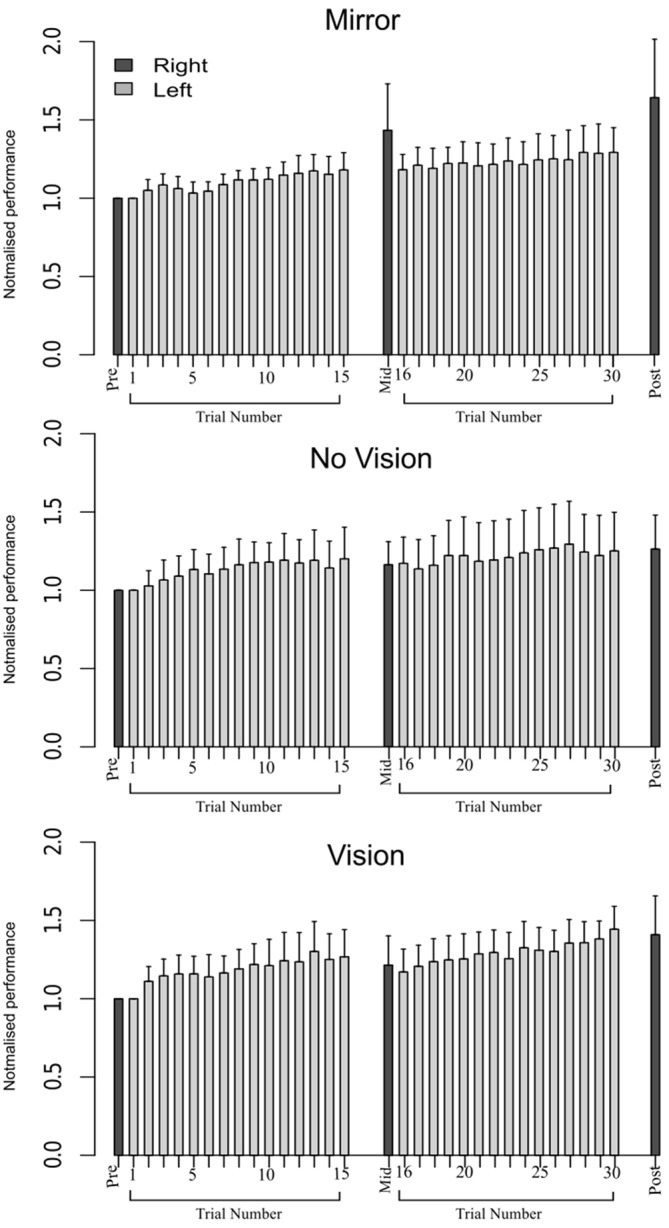
**Experiment 1.** Left (light gray shading) and right limb (dark gray shading) performance data are shown normalized to the respective baseline values (i.e., the mean peak acceleration recorded during the initial trial). Each trial comprised 10 movements. The error bars correspond to 95% confidence intervals (CI) calculated across participants. Note that in the initial trials, the values for all participants are equal to one. The data are shown separately for participants in the “Mirror”, “No Vision” and “Vision” conditions.

In order to eliminate participants who did not engage fully with the training task in the fashion intended, we excluded from further analysis those who failed to exhibit improvements in performance (i.e., changes in the peak accelerations of the ballistic movements exhibited for the training (left) limb) above a criterion defined empirically by the adjusted boxplot method for outlier rejection. This method, when applied to values pooled across the three vision conditions, revealed the presence of one outlier (5.89%) falling below a threshold criterion of 14% improvement. With the outlier removed, independent equivalence tests (Wellek and Michaelis, 1991) were carried out to establish whether the visual feedback groups differed with respect to the improvements in performance of the training limb (Mirror: *Mdn* = 36.84%, *IQR* = 18.55–54.38, No Vision: *Mdn* = 27.88%, *IQR* = 20.76–45.90, Vision: *Mdn* = 48.33%, *IQR* = 33.83–62.48) derived from the three hundred movement repetitions (Figure 4). As these data were not normally distributed, a log transform was first applied to increase the symmetry of the sample distribution. All equivalence tests were performed on the basis of the log transformed data. On the basis of an Epilson value of 0.72, the training related improvements in performance were deemed equivalent for the Mirror and No Vision group (*p* = 0.008), and for the Mirror and Vision group (*p* = 0.04). The corresponding contrast between the No Vision and Vision groups approached conventional levels of statistical significance (*p* = 0.07). Thus any differences observed between the groups in terms of the degree of transfer, cannot reasonably be attributed to variations in the improvements in performance of the left limb derived from training.

**Figure 5:**
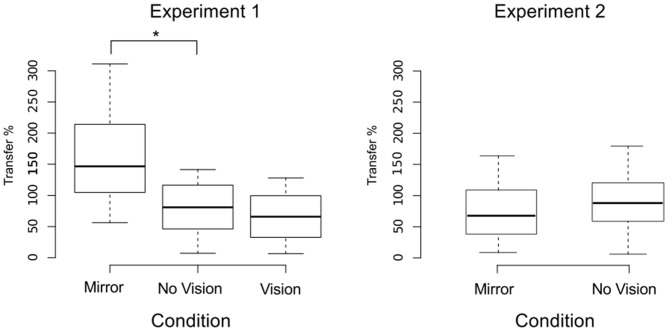
**Transfer of performance.** The magnitude of interlimb transfer was calculated as the change in performance of the untrained (right) limb, expressed as a percentage of the change in performance of the (left) training limb. Separate boxplots are shown for the “Mirror”, “No Vision” and “Vision” groups in Experiment 1, and for the “Mirror” and “No Vision” groups in Experiment 2. The horizontal band within each box represents the median value. The bottom and top of the boxes are the first and third quartiles respectively. The whiskers correspond to the lowest datum within 1.5 IQR (inter-quartile range) of the first quartile, and the highest datum within 1.5 IQR of the third quartile. Braces marked with the “*” symbol represent instances in which a difference between two groups in the degree of transfer was statistically reliable.

We also sought to exclude participants who exhibited changes in the performance of the untrained (right) limb sufficiently distant from the other observations that they could be considered drawn from a population outside that being examined. In order to do so, an exclusion criterion was defined empirically using the adjusted boxplot method for outlier rejection. With respect to the variations in the peak accelerations of the ballistic movements exhibited for the untrained limb, this method revealed the presence of two outliers falling below a threshold criterion of 0% improvement. The magnitude of interlimb transfer was calculated for the remaining participants as the change in performance of the untrained (right) limb, expressed as a percentage of the change in performance of the training limb. A Shapiro-Wilk test of normality revealed that the resulting distribution of transfer values was not normal (*W* = 0.93, *p* = 0.048). Pairwise contrasts were hence conducted using Mann Whitney *U* tests, comparing the Mirror group (*Median* = 146.5%; *CI* = 102.3–214.1%) to the (control) No Vision group (*Median* = 81.0%; *CI* = 16.4–102.0%), and the Vision group (*Median* = 52.3%; *CI* = 19.3–89.1%) to the (control) No Vision group. This revealed that the level of transfer was greater in the Mirror condition than in the No Vision condition (*U* = 92, df = 19, *p* = 0.007, *r* = 0.45; Figure 5).

#### Measures of Corticospinal Excitability

For each visual feedback condition, planned contrasts based on mixed effects models were conducted to compare the AURC values obtained prior to training (pre), with those obtained at the mid point of training (mid), and following (post) training. Separate analyses were carried out for FCR and ECR, and for the right and left limbs. As the raw AURC values were not normally distributed, they were first subject to a log transformation. Fitting of the mixed effects models employed restricted maximum likelihood (REML) estimation and an unstructured covariance matrix. Participants crossed with time (levels = pre, mid, post) was designated a random effect. Time was a fixed effect. In order to assist in the interpretation of the inferential analyses, effect size indices (Cohen’s *d*) were calculated (Cohen, 1988). By convention, an effect size of 0.2 is considered small, 0.50 is moderate and a value of 0.8 or above is considered to be large.

**Figure 6:**
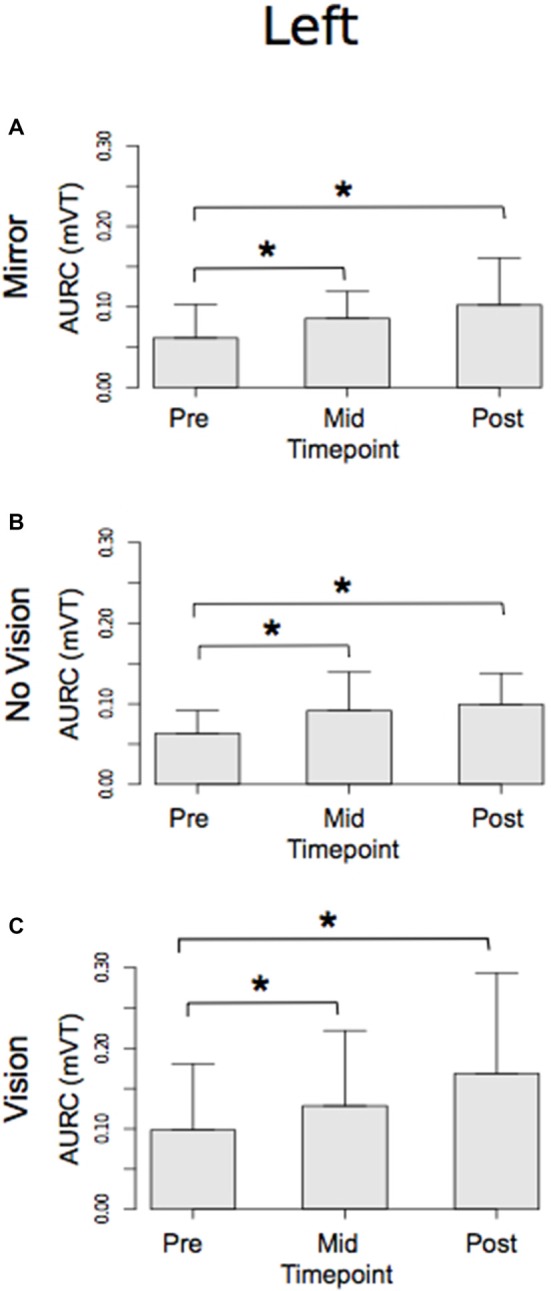
**Experiment 1.** Measures of CSE for the left FCR are shown separately for the “Mirror”, “No Vision” and “Vision” visual feedback conditions. **(A–C)** Represent the mean area under the recruitment curve (AURC)—a measure of CSE. These recordings were obtained prior to (“pre”), at the midpoint of (“mid”—commencing 5 min following trial 15), and following (“post”—commencing 5 min following trial 30) the training undertaken by the left limb. Braces marked with the “*” symbol represent instances in which a difference between two measurements was statistically reliable. The error bars correspond to 95% CI for repeated measures designs, calculated following Cousineau (2005) and Morey (2008).

**Figure 7:**
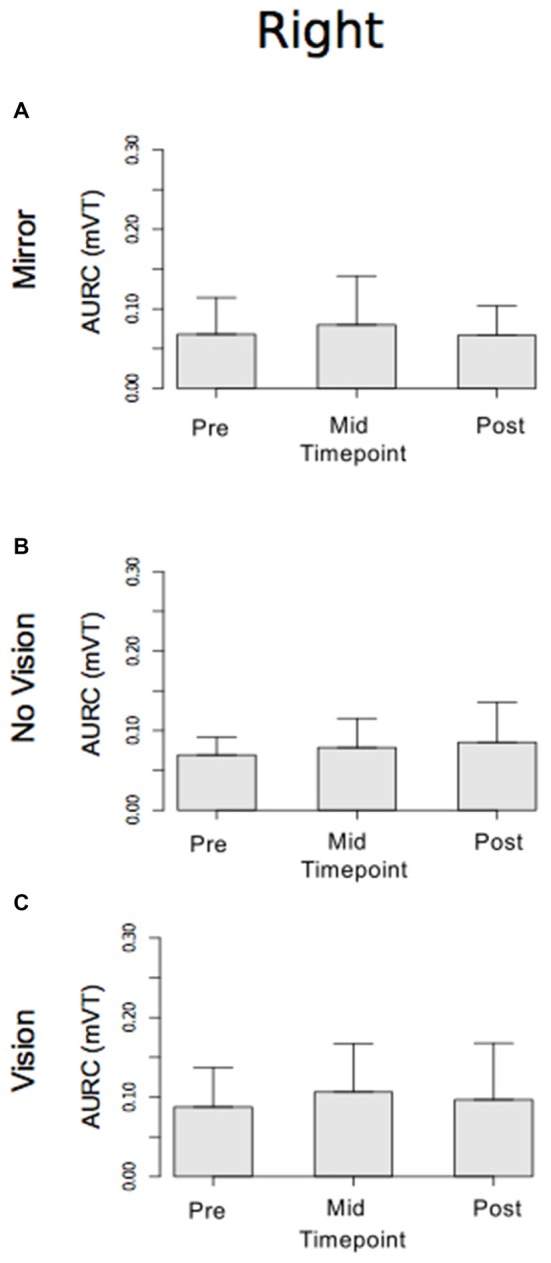
**Experiment 1.** Measures of CSE for the right FCR are shown separately for the “Mirror”, “No Vision” and “Vision” visual feedback conditions. **(A–C)** Represent the mean area under the recruitment curve (AURC)—a measure of CSE. These recordings were obtained prior to (“pre”), at the midpoint of (“mid”—commencing 10 min following trial 15), and following (“post”—commencing 10 min following trial 30) the training undertaken by the left limb. The error bars correspond to 95% CI for repeated measures designs, calculated following Cousineau (2005) and Morey (2008).

With respect to the (left) limb that performed the three hundred training movements, pronounced increases in the AURC for the agonist FCR muscle were detected for all three visual feedback conditions when assessed during the interval between the two blocks of training (>5 min following Trial 15), and following the termination of training (>5 min following Trial 30; Figure 6). In marked contrast, no reliable changes in the AURC for the contralateral homolog (right FCR) of the opposite limb were present for any of the visual feedback conditions (Table 1 and Figure 7).

**Table 1:** **Experiment 1: Pairwise comparisons between AURC values obtained prior to (Pre) and following (Post) training are presented for the left and right FCR and ECR, in the Mirror, No Vision and Vision conditions**.

Condition	Muscle	*t* (*df*)	*p* value	*d*
No Vision	LFCR	4.085 (20)	**0.001**	1.757
	LECR	3.017 (20)	**0.007**	1.298
	RFCR	1.866 (20)	0.077	0.803
	RECR	2.244 (20)	**0.036**	0.965
Mirror	LFCR	2.928 (18)	**0.009**	1.322
	LECR	0.592 (18)	0.561	0.267
	RFCR	−0.099 (18)	0.922	0.045
	RECR	0.357 (18)	0.725	0.161
Vision	LFCR	5.481 (22)	**<0.001**	2.257
	LECR	0.543 (22)	0.593	0.223
	RFCR	0.591 (22)	0.561	0.243
	RECR	1.632 (22)	0.117	0.672

*Statistically reliable (p < 0.05) pairwise differences are highlighted in bold font. Cohen’s index of effect size (d) is also given*.

A reliable change in the AURC for the left ECR was observed in only one instance. In the No Vision condition, the AURC values obtained post-training were higher than those recorded prior to training (Table 1). In relation to the right ECR, there were no reliable changes in AURC evident for the Mirror condition. In the vision condition, AURC values obtained during the interval between the two blocks of training were higher than those recorded prior to training, and in the No Vision condition a similar change was expressed following the termination of training (Table 1). We will return to the possible origin of the latter changes in the sections that follow.

### Experiment 2

#### Behavioral Outcomes

With a view to ensuring comparability, the screening criteria applied in Experiment 1 (based on the adjusted boxplot method of outlier detection) were applied to the second dataset. This resulted in the removal of 12 participants on the basis of failure to demonstrate improvements in performance of the training limb greater than 14%, and 11 participants due to improvements in performance of the right limb that were below 0%. As it is likely that this screening method primarily excludes participants who did not fully engage with the task, it is unsurprising that many of these “outliers” were eliminated on both grounds. The total number of participants removed was 18 (63 remaining).

**Figure 8:**
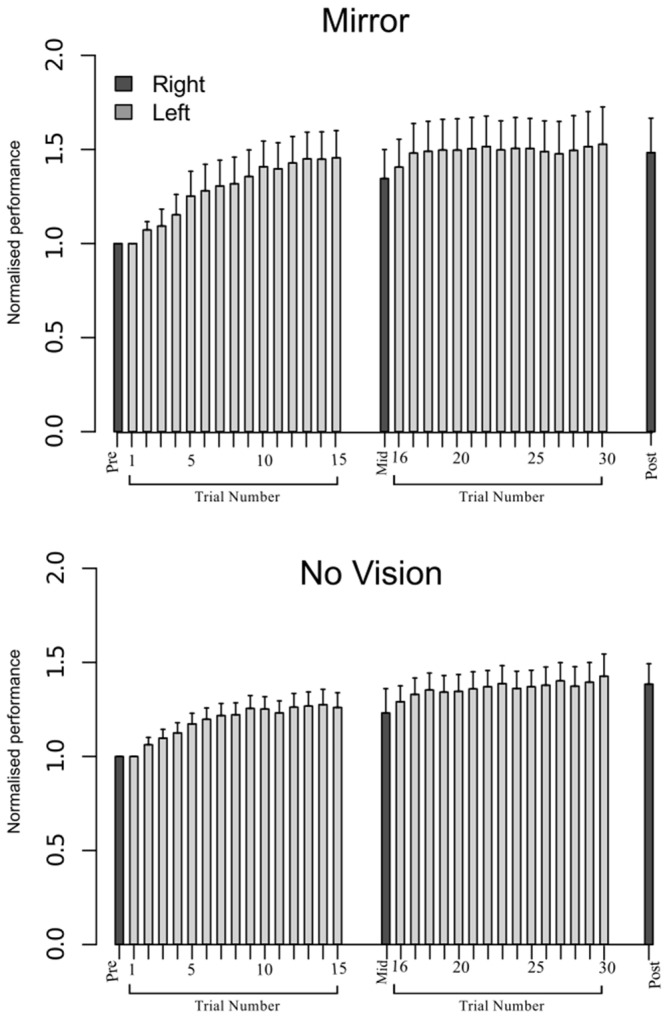
**Experiment 2.** Left (light gray shading) and right limb (dark gray shading) performance data are shown normalized to the respective baseline values (i.e., the mean peak acceleration recorded during the initial trial). Each trial comprised 10 movements. The error bars correspond to 95% CI calculated across participants. Note that in the initial trials, the values for all participants are equal to one. The data are shown separately for participants in the Mirror and No Vision conditions.

An equivalence test applied to the log-transformed performance data confirmed that for the left limb, the increases in peak acceleration due to training were comparable for the two groups (Mirror: *Mdn* = 52.17%, No Vision: *Mdn* = 39.68%, *p* = 0.003; Figure 8). Transfer was calculated in the manner described for Experiment 1, and resulted in a distribution of values that deviated from normality (Shapiro Wilk test: *W* = 0.93, *p* = 0.002). In contrast to the outcome obtained in Experiment 1, the level of transfer present in the Mirror condition (*Median* = 67.8%; *CI* = 42.8–83.0%) and the No Vision condition (*Median* = 88.1%; *CI* = 71.5–103.7%) did not differ reliably (Mann Whitney *U* = 653, df = 61, *p* = 0.16; Figure 5).

#### Relationships Between Variations in Left and Right Limb Performance

To establish whether there were relationships between the changes in performance exhibited by the left and right limbs in the two visual feedback conditions, statistical measures of association (correlation and regression) were derived. As it is well documented that least squares variants of these measures are extremely sensitive to the presence of univariate and bivariate outliers, robust methods (e.g., Wilcox, 2012) were used in all cases. We note that such outliers may be present in circumstances in which the respective sample distributions are deemed normally distributed. Confidence intervals (95%) derived using a bootstrapping approach are reported for both measures.

As is evident from inspection of the values presented in Table 2, in both conditions there was a reliable positive association between the change in performance of the training limb (i.e., the peak acceleration of the trial wherein they performed best, expressed relative to the peak acceleration of trial 1), and the change in performance exhibited for the untrained (right) limb (i.e., expressed relative to the peak acceleration achieved prior to the onset of training).

**Table 2:** **Experiment 2: Measures of association between: changes in performance of the training limb (i.e., the peak acceleration of the trial wherein they performed best expressed relative to the peak acceleration of trial 1), and changes in performance exhibited for the untrained (right) limb (i.e., expressed relative to the peak acceleration achieved prior to the onset of training); changes in performance exhibited by the (left) training limb and levels of transfer**.

Δ Left ~ Δ Right	Measure	*n/df*	*r*	*Slope*	*t*	*p*	95% CI
Mirror	Correlation	31	0.54	–	3.49	**0.002**	0.24–0.77
Mirror	Regression	29	–	0.23	1.68	0.10	0.01–0.94
No Vision	Correlation	34	0.64	–	4.73	**<0.001**	0.39–0.84
No Vision	Regression	32	–	0.87	2.99	**0.005**	0.49–1.52

**Δ Left ~ Transfer**	**Measure**	***df***	***r***	***Slope***	***t***	***p***	**95% CI**

Mirror	Correlation	31	−0.03	–	0.19	0.85	−0.42–0.31
Mirror	Regression	29	–	−0.07	0.49	0.63	−0.43–0.38
No Vision	Correlation	34	−0.03	–	0.17	0.87	−0.36–0.35
No Vision	Regression	32	–	0.21	1.08	0.29	−0.87–0.56

#### Relationships Between Variations in Left Limb Performance and Transfer

We also sought to establish whether there were reliable associations between the level of transfer and the changes in the peak accelerations of the ballistic movements exhibited by the (left) training limb. In marked contrast to the relationship that characterized the changes in performance exhibited by the left and right limbs, the magnitude of interlimb transfer (i.e., the *degree* of benefit accrued from training of the opposite limb) was not associated with the magnitude of the gains in performance achieved for the training limb (Table 2).

#### Measures of Corticospinal Excitability

**Figure 9:**
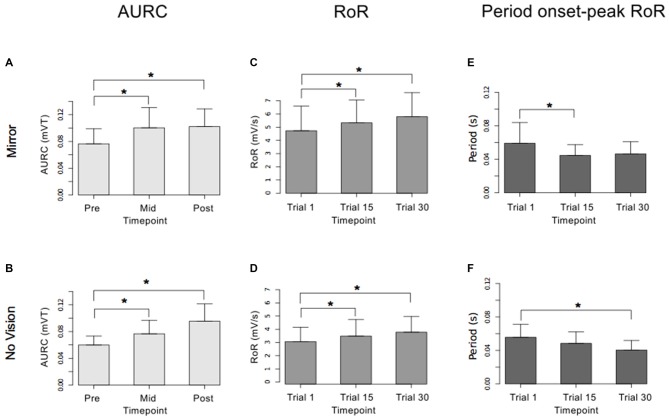
**Experiment 2.** Measures of CSE and muscle activation dynamics for the left FCR are shown separately for the “Mirror” and “No Vision” visual feedback conditions. **(A,B)** Represent the mean area under the recruitment curve (AURC)—a measure of CSE. These recordings were obtained prior to (“pre”), at the midpoint of (“mid”—commencing 5 min following trial 15), and following (“post”—commencing 5 min following trial 30) the training undertaken by the left limb. **(C,D)** Represent the mean maximum rate of rise (RoR) of the left FCR EMG. **(E,F)** Represent the mean period from the onset of EMG activity in left FCR to the time at which the maximum RoR occurred. These measurements (mean RoR and mean time from onset to maximum RoR) were derived from training trials 1, 15 and 30. Braces marked with the “*” symbol represent instances in which a difference between two measurements was statistically reliable. The error bars correspond to 95% CI for repeated measures designs, calculated following Cousineau (2005) and Morey (2008).

**Figure 10:**
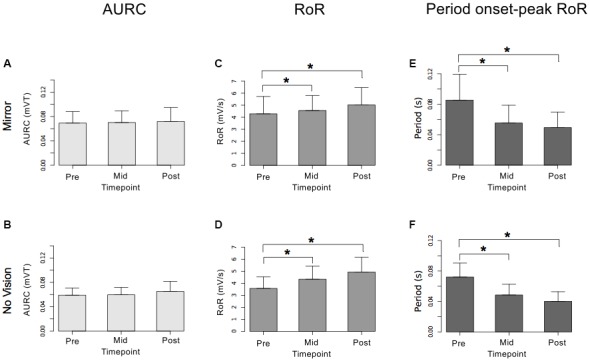
**Experiment 2.** Measures of CSE and muscle activation dynamics for the right FCR are shown separately for the “Mirror” and “No Vision” visual feedback conditions. **(A,B)** Represent the mean area under the recruitment curve (AURC)—a measure of CSE. **(C,D)** Represent the mean maximum rate of rise (RoR) of the right FCR EMG. **(E,F)** Represent the mean period from the onset of EMG activity in right FCR to the time at which the maximum RoR occurred. These recordings were obtained prior to (“pre”), at the midpoint of (“mid”—commencing 10 min following trial 15), and following (“post”—commencing 10 min following trial 30) the training undertaken by the left limb. Braces marked with the “*” symbol represent instances in which a difference between two measurements was statistically reliable. The error bars correspond to 95% CI for repeated measures designs, calculated following Cousineau (2005) and Morey (2008).

In both visual feedback conditions, when assessed during the interval between the two blocks of training, and following the termination of training, reliable increases in the AURC for the left FCR (i.e., the training limb) were detected (Table 3 and Figure 9). No changes in the AURC of the contralateral homolog (right FCR) of the untrained limb were present for either of the visual feedback conditions (Table 3 and Figure 10). Indeed, in the Mirror condition, the AURC values obtained following training and at the mid point of training were statistically equivalent to those recorded prior to training. This was also the case in the No Vision condition for the AURC values recorded during the interval between the two blocks of training.

**Table 3:** **Experiment 2: Pairwise comparisons between AURC values obtained prior to (Pre) and following (Post) training are presented for the left and right FCR and ECR, in the Mirror and No Vision conditions**.

Condition	Muscle	*t* (*df*)	*p* value	*d*
No Vision	LFCR	8.220 (68)	**<0.001**	1.971
	LECR	1.806 (68)	0.075	0.433
	RFCR	1.543 (68)	0.127	0.370
	RECR	3.585 (68)	**0.001**	0.860
Mirror	LFCR	5.892 (60)	**<0.001**	1.502
	LECR	0.042 (60)	0.966	0.011
	RFCR	−0.015 (60)	0.988	0.004
	RECR	0.049 (60)	0.961	0.012

*Statistically reliable (p < 0.05) pairwise differences are highlighted in bold font. Cohen’s index of effect size (d) is also given*.

A reliable change in the AURC for the left ECR was observed in only one instance. In the Mirror condition, the AURC values obtained at the midpoint of training were higher than those recorded prior to training (Table 3). With respect to the right ECR, in the No Vision condition, AURC values recorded following training and at the mid point of training were larger than those prior to training (Table 3).

#### Relationships Between Variations in Corticospinal Excitability and Transfer

In reporting these analyses, we take the opportunity to highlight the following. The absence of a net change in CSE (AURC)—as calculated across participants (i.e., between pre- and post-training assessments), does not preclude the possibility of a reliable association between the change in CSE exhibited by each individual (positive or negative) and the level of transfer they exhibited. There was however no evidence of associations between the degree of interlimb transfer manifested by individual participants, and the pre- to post-training change in the right FCR AURC in either of the visual feedback conditions (associated *p* values 0.09–0.95).

#### Measures of Muscle Activation Dynamics

For the limb that performed the training movements, in both visual feedback conditions, the mean maximum RoR of the left FCR EMG obtained during trial 15 and during trial 30 was greater than the mean value derived for trial 1 (Figure 9). For the untrained (right) limb, in both visual feedback conditions, the mean maximum RoR of the FCR EMG obtained during the Mid trial—which commenced 10 min following training trial 15, and during the Post trial—which commenced 10 min following training trial 30, were markedly larger than the mean value calculated for the Pre trial (Figure 10 and Table 4).

**Table 4:** **Experiment 2: Pairwise comparisons between the maximum RoR of right FCR EMG obtained prior to (Pre) and following (Post—commencing 10 min following trial 30) training of the opposite limb, and the maximum RoR of left FCR EMG (i.e., the training limb) obtained during trial 1 and trial 30, in the Mirror and No Vision conditions**.

Condition	Limb	*df*	*t* (RoR)	*p* (RoR)	*d* (RoR)	*t* (period)	*p* (period)	*d* (period)
No Vision	Right (Pre-Post)	62	8.548	**<0.001**	1.321	−7.946	**<0.001**	1.994
Mirror	Right (Pre-Post)	60	3.705	**<0.001**	0.945	−4.879	**<0.001**	1.244
No Vision	Left (Trial 1 vs. 30)	62	5.579	**<0.001**	1.400	−3.447	**0.001**	0.865
Mirror	Left (Trial 1 vs. 30)	60	5.840	**<0.001**	1.489	−1.920	0.060	0.489

*Corresponding contrasts for the period from the onset of EMG activity in FCR to the time at which the maximum RoR occurred are also presented. Statistically reliable (p < 0.05) pairwise differences are highlighted in bold font. Cohen’s index of effect size (d) is also given*.

In both visual feedback conditions, there was evidence to suggest that the period from the onset of left FCR EMG activity to the time of the maximum RoR was shorter during trial 30 than during trial 1 (Table 4). For the untrained right limb, conspicuous decreases in the time to the maximum RoR were apparent in both visual feedback conditions, when assessed during the interval between the two blocks of left limb training, and (10 min) following the cessation of training by the opposite limb (Figure 10 and Table 4).

#### Relationships Between Variations in Muscle Activation Dynamics and Transfer

In light of the close similarity of the changes in muscle activation dynamics obtained for the untrained limb in the Mirror and No Vision conditions, and with a view to increasing statistical power, the data were pooled for the purposes of calculating measures of association. This was the only instance in which data were pooled. As we wished to focus upon the possibility that the observed variations in muscle activation dynamics have explanatory power beyond their obligatory association with the performance of the untrained limb, partial correlations were calculated, whereby the variance attributable to changes in the performance of the untrained limb was removed. To similarly ensure that the measures of associations were not directly contingent upon the magnitude of the gains in performance achieved for the training limb, the variance attributable to this factor was also removed.

**Figure 11:**
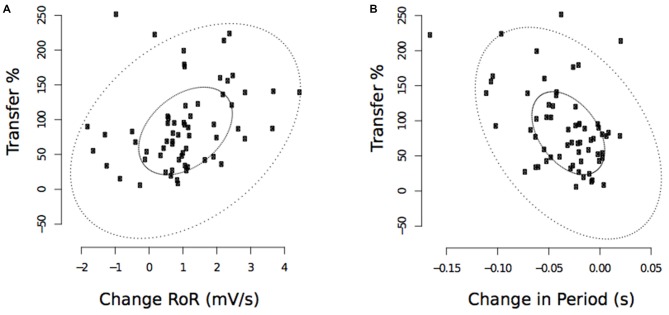
**Experiment 2.** Associations between changes in the maximum RoR of right FCR EMG and the degree of transfer, and between changes in the period from the onset of right FCR EMG activity to the time of the maximum RoR and the degree of transfer. **(A)** Corresponds to maximum RoR values obtained following the cessation of training undertaken by the opposite limb (“post”—commencing 10 min following trial 30). **(B)** Corresponds to period values. The smaller ellipse contains half of the data. Points outside the larger ellipse are considered to be outliers.

These analyses revealed that the observed decreases in the period from the onset of right FCR EMG activity to the time of the maximum RoR assessed following the cessation of training, were negatively correlated with the level of transfer (r(PB) = −0.46, *t*_(61)_ = 2.31, *p* < 0.01; CI(95%) = −0.65 to −0.28; Figure 11). The pre to post training changes in the maximum RoR of the right FCR EMG were positively correlated with the degree of transfer, albeit to a degree that was not statistically reliable (*r*_(PB)_ = 0.22, *t*_(61)_ = 1.78, *p* = 0.08; CI(95%) = −0.05 to 0.52; Figure 11).

#### FCR-ECR Co-Contraction

For the training limb, the level of ECR co-contraction (during FCR bursts) present in trial 15 or trial 30, was not different from that exhibited in trial 1. This was the case for both visual feedback conditions (Mirror: Trial 1 *Mdn* = 0.23, Trial 30 *Mdn* = 0.18, No Mirror: Trial 1 *Mdn* = 0.25, Trial 30 *Mdn* = 0.26). Similarly, the levels of co-contraction exhibited during movements of the untrained limb were not altered following training (Mirror: Pre *Mdn* = 0.19, Post *Mdn* = 0.21, No Mirror: Pre *Mdn* = 0.19, Post *Mdn* = 0.19). Levels of FCR-ECR co-contraction were not reliably correlated with, or predictive of, transfer in either of the visual feedback conditions (associated *p* values 0.08–0.96).

#### Activity Detected in Right FCR and ECR during (Left Limb) Training Movements

Detectable bursts of EMG activity in the FCR of the non-moving right limb were present during 43% (median) of training movements performed in the Mirror condition (*IQR* = 15.72–68.26), and during 26.04% (median) of trials in the No Vision condition (*IQR* = 10.33–65.13. The two conditions were not differentiated in this regard (*U* = 439, *df* = 61 *p* = 0.44). Furthermore, the frequency with which detectable bursts of EMG activity were present in the right FCR during training movements of the opposite limb was not correlated with, or predictive of, transfer in either of the visual feedback conditions (associated *p* values 0.08–0.32).

Robust regressions additionally revealed that the frequency of right FCR burst activity (during left limb training movements) was a not reliable predictor of pre- to post or pre-mid training changes in the AURC measures obtained for this muscle (associated *p* values 0.52–0.96).

With respect to the right ECR, bursts were detected in 22.48% of trials in the Mirror condition (*IQR* = 4.17–55.80), and during 20.23% of trials in the No Vision condition (*IQR* = 5.94–33.20). There were no reliable differences between conditions (*U* = 483.5, df = 61, *p* = 0.87). The frequency of right ECR burst activity (during left limb training movements) was not correlated with or predictive of pre to mid or pre- to post-training changes in the AURC measures obtained for this muscle (associated *p* values 0.14–0.90).

The mean of the relative phase relationship between the derivatives of the respective left and right FCR enveloped EMG signals was −12.94**°** (347.06°) in the Mirror condition, and −18.87° (341.13°) in the No-Vision condition (in both cases activity in left FCR was on average in advance of activity in right FCR). The uniformity of this relationship was greater in the Mirror condition (*Mdn* = 0.49, *IQR* = 0.42–0.66) than in the No-Vision condition (*Mdn* = 0.40, *IQR* = 0.38–0.50. *U* = 671, *p* = 0.02). The uniformity of the relative phase relation was not however correlated with, or predicative of, transfer in either of the visual feedback conditions (associated *p* values 0.09–0.90).

## Discussion

The repetition of 300 ballistic movements, each executed with the intent of maximizing acceleration, gave rise to substantial increases in the performance of the opposite limb. The median level of interlimb transfer (i.e., change in performance of the untrained limb, expressed as a percentage of the change in performance of the training limb) exhibited by the 96 participants retained in our analyses (i.e., drawn from the two datasets) was 82.44%. This high degree of cross education is consistent with the outcomes of previous studies in which tasks requiring maximal motor output have been employed (Zhou, 2000; Carroll et al., 2006). Since *during* the unilateral execution of such training tasks there is an acute increase in the excitability of both contralateral and ipsilateral corticospinal projections (Carson, 2005), it has been proposed previously that interlimb transfer of performance may be mediated by interactions between the primary motor cortices (e.g., Hinder et al., 2011). To examine this possibility, we manipulated visual feedback of the limb that executed the training movements—a factor that modulates the excitability of descending projections from M1 to the homologous muscles of the opposite limb (Carson et al., 2005; Garry et al., 2005; Carson and Ruddy, 2012).

In the first of two experiments, we observed that the magnitude of transfer was greater in a condition in which the participants viewed and attended to the mirror image of the training limb, than in two other conditions in which they either looked at the non-moving limb or received no movement-related visual feedback. In a second experiment that engaged a larger number of participants, this finding was not replicated. More tellingly, in neither experiment were there instances in which the increased capability of the untrained limb was accompanied by changes in the excitability of corticospinal projections to a muscle which acted as a principal agonist for the wrist flexion task. Furthermore, there was no evidence to suggest that for individual participants increases in the excitability of these projections was positively associated with, or predictive of, the degree of cross education that was achieved.

There have been previous reports of increases in the excitability of corticospinal projections to the untrained limb (at rest) in the period following acute (e.g., Carroll et al., 2008; Hinder et al., 2011; Poh et al., 2013; Reissig et al., 2015) and chronic (Hortobágyi et al., 2011) training. The foregoing studies differed from the present investigation in a number of respects. Firstly, the movements under consideration were abductions of the index finger—an action to which the first dorsal interosseus (an intrinsic hand muscle) makes a significant functional contribution. Secondly, the dominant (typically right) limb performed the training movements. Furthermore, in at least some of these studies, measurements of CSE were obtained when less than 5 min had elapsed following the cessation of training. These reported increases in CSE at rest notwithstanding, we are aware of no instances in which such changes have been shown to correlate with, or predict, the magnitude of interlimb transfer that is exhibited in tasks demanding maximum motor output.

It is believed that TMS over M1 exerts an effect on chains of interneurons with fixed temporal characteristics that produce a periodic bombardment of corticospinal neurons (Amassian et al., 1987). When, as in the present study, the direction of the induced brain current is (initially) posterior to anterior, the successive components of motor evoked potentials elicited at threshold intensities (by both monophasic and biphasic pulses) are thought to first reflect the activation of corticocortical axons projecting onto corticospinal neurons, or axon collaterals of corticofugal systems (e.g., motor areas such as dorsal premotor cortex (PMd), ventral premotor cortex (PMv) and supplementary motor area (SMA)) with corresponding projections i.e., onto corticospinal neurons (Di Lazzaro et al., 2008), and subsequently transmission via polysynaptic networks or recurrent synaptic networks (cf. Rusu et al., 2014). As the intensity of stimulation is increased (to 150% RMT in the present study) in generating a recruitment curve, neurons in addition to those activated at threshold, which are intrinsically less excitable or are spatially removed from the peak of the magnetic field, also contribute to the descending volley. The AURC measure employed in our experiments thus broadly represents the post-synaptic state of the subpopulation of (large diameter, fast conducting) pyramidal tract neurons activated by TMS, and of the local networks—presumed to be predominantly within M1, that are presynaptic to these cells. On this basis we are confident in concluding that with respect to ballistic (“fast as possible”) movement training, there is no evidence to support the conjecture that interlimb transfer of performance is mediated by alterations in the resting state of these specific neural elements.

While the elevated level of transfer exhibited by participants in the Mirror condition in Experiment 1 was not obviously attributable to changes in the state of the neural elements activated at rest by TMS, the performance enhancement of the untrained limb was nonetheless striking (amounting to a 55.7% increase in peak acceleration). Although the effect was not expressed for the larger samples used in the second experiment, it is worth remarking that in this latter case the four participants demonstrating the highest levels of transfer were drawn from the group assigned to the Mirror condition. Individuals demonstrate large variation in their capacity for motor imagery (Gregg et al., 2010; Williams et al., 2012), and it is likely that such variations extend also to the impact of mirrored visual feedback (Mercier and Sirigu, 2009). It is possible that in the context of the relatively small groups employed in Experiment 1 (*n* = 12), there was for the Mirror condition an overrepresentation (relative to the general population) of individuals predisposed to respond positively to the provision of this form of visual feedback. While the results thus provide only partial support for the hypothesis that by attending to a mirror image of the moving limb the degree of transfer to the untrained limb will be accentuated (Howatson et al., 2013; Zult et al., 2014), the degree to which individuals vary in the extent to which such feedback augments the acquisition of motor skill (e.g., Nojima et al., 2012) remains to be determined. Indeed, as in relation to the mechanisms that mediate cross education more generally (Ruddy and Carson, 2013), the benefits accrued from augmented sensory feedback are likely to be highly task dependent.

It has been shown previously that the magnitude of the increases in functional capacity exhibited for the non-training limb, are related to the gains accrued by the training limb (Zhou, 2000). The outcomes of the present study are in accordance with these previous observations, whereby there were positive associations (across participants) between the changes in performance of the training limb and the changes in performance exhibited for the untrained limb. This is also in accordance with intuition, since variations between individuals with respect to such factors as motivation, level of engagement, and susceptibility to central fatigue, will presumably impact equivalently upon the performance of both limbs. It is particularly notable therefore that the level of cross education (i.e., the *degree* of (proportional) benefit accrued from training of the opposite limb) was not associated with the magnitude of the gains in performance achieved for the training limb. This result suggests that there are mechanisms mediating the transfer of functional capacity between the limbs, which are at least partially dissociable from those that engender generalized increases in performance evident for both limbs, and that they vary across individuals in terms of their efficiency.

The entirely novel finding arising from the present study was the presence of an association between alterations in activation dynamics (i.e., time to maximum RoR)—following training performed by the opposite limb, and the level of *transfer* that arose from that training. Importantly, this association was evident when variance attributable to the gains in performance achieved for both the training limb and the untrained limb was removed. Given the association with the “transfer gain”, it seems reasonable to conclude that the decreases in time to maximum RoR of the agonist EMG, are an expression of at least some of the acute neural adaptations that constitute the basis of cross education in this task.

The observed association between alterations in muscle activation dynamics and the level of transfer, prompts consideration of other cortical regions including SMA and anterior cingulate cortex (ACC), that are thought to play an instrumental role in the *initiation* of movement (Deecke and Kornhuber, 1978; Hoffstaedter et al., 2012). It has been proposed previously that SMA mediates the relationship between generalized internal drive manifested through ACC, and the specification of motor commands instantiated via circuits within M1 (Goldberg, 1985). SMA exhibits dense connectivity with cortical and subcortical motor structures and by virtue of ipsilateral and contralateral projections, it has the potential to both influence control of the contralateral limb through fibers reaching ipsilateral M1, and modulate the influence of the opposite SMA through callosal connections to the contralateral SMA (and M1; Goldberg, 1985).

Electromyographic activity recorded in the muscles of the non-moving limb during the execution of training movements, although commonly reported, is not believed to reflect neural processes that play a causal role in relation to interlimb transfer of performance (Carroll et al., 2006). In the experiments described herein, this “mirrored” EMG activity was quantified in two ways: by enumeration of detectible bursts; and by calculating the uniformity of the relative phase relationship between the electromyograms recorded simultaneously from left and right FCR. With respect to neither measure was there an association with transfer, further reinforcing the view that the contralateral irradiation (Cernacek, 1961) is an epiphenomenon that does not bear directly upon the improvements in performance observed for the untrained limb. There were a few instances in which an increase in the excitability of corticospinal projections to the ECR of the untrained limb was observed following training. For the Vision group in Experiment 1, and the No-Vision group in Experiment 2, there was an association between the extent of this increase, and the frequency with which EMG bursts were detected in the muscle during the preceding training movements. It is possible that the ECR muscle activity present during the (maximal effort) movements of the opposite limb simply reflects the demands of postural stabilization, in which context the persisting increases in the excitability of descending projections to the muscle are a natural consequence.

The results of the current investigation suggest that the circuits within primary M1 that are recruited at rest by single-pulse TMS (the initial phase inducing posterior to anterior current flow in the brain) do not constitute the primary locus of the neural adaptations that mediate interlimb transfer of the performance gains realized through unilateral practice of this ballistic motor task. The majority of the short latency responses to TMS evoked in healthy adults are however mediated by large corticospinal neurons with fast-conducting axons (Lemon, 2002). Slower conducting axons also make monosynaptic connections with upper limb motoneurons (Porter and Lemon, 1995) and it remains possible that cortical circuits projecting onto these cells exhibit distinct patterns of adaptation in response to training.

Beyond the recognition that TMS is capable of sampling only a subset of circuits in primary M1, it is also becoming apparent that the intensity of stimulation, and orientation of the induced current flow determines the composition of this subset—in terms of the balance of inhibitory and excitatory inputs to the corticospinal output cells (Di Lazzaro and Rothwell, 2014). Different populations of fibers are likely to be excited by anterior-posterior (AP) as opposed to PA currents (Di Lazzaro et al., 2001). Although the most probable neuronal site for activation by TMS is at the fiber terminal, it is also possible that large afferent axons from premotor and somatosensory areas may be especially sensitive to AP currents (Esser et al., 2005). These fibers constitute the main cortical input to M1 (DeFelipe et al., 1986; Sutor et al., 2000). The induction of current flows in directions other than posterior to anterior may therefore reveal variations in the state of projections from other elements of the motor network onto targets within M1. Necessarily however, a comprehensive appraisal of the putative role of primary M1 in mediating interlimb transfer of motor function requires additional experimental techniques, extending beyond those based on TMS.

The observed associations between alterations in muscle activation dynamics, and the degree of transfer that arose from training of the opposite limb, suggest adaptations occurring functionally upstream of the neural elements within primary M1 that are recruited at rest by single-pulse TMS. Nonetheless, it is possible to conceive of a number of other means through which alterations in muscle activation dynamics may be mediated. In the context of tasks demanding maximum motor output, elevations in the excitability of the spinal motoneuron pool may be realized via descending pathways from other parts of the cortical motor network. At least in monkey, there are direct corticospinal projections onto spinal motoneurons from supplementary and premotor areas (Dum and Strick, 1996, 2002) and from parietal regions (Murray and Coulter, 1981). In principle, a component of the descending motor command may also be mediated through propriospinal relays, potentially subject to the influence of reticulospinal projections (Pierrot-Deseilligny, 1996; Rothwell, 2002).

The foregoing considerations suggest that generalizations concerning the relationship between changes in MEP amplitude and the processes of motor learning should be made with caution (Carson et al., in press). While it may be the case that the performance of tasks that give rise to motor learning is accompanied by systematic and reliable changes in CSE, evidence of an instrumental relationship between the degree of change in CSE, and the learning that accrues to an individual, remains extremely sparse (Ljubisavljevic, 2006). This is not to imply that the primary M1 is not intimately involved in processes underlying motor learning. Indeed, widespread evidence derived from neuroimaging suggests that M1 is integral to a network of brain regions involved in the learning and retention of motor skills. It is nonetheless the case that context-dependent variations in these processes and their balance, are challenging to resolve using the tools currently available in human electrophysiology. There are other issues that bear contemplation. Primary M1 contributions to motor learning in general, and to interlimb transfer of learning in particular, are likely to vary in a task and time-contingent fashion. Necessarily the same can be said of all elements of the motor network (Ruddy and Carson, 2013). The results of the present investigation should be interpreted in this context. They provide a partial representation of complex multifactorial and multilevel adaptive mechanisms bound by a specific experimental context.

The interlimb transfer of functional capacity that is expressed in the context of a ballistic movement task is mediated by neural elements other than those within primary M1 that are recruited at rest by (PA) single-pulse TMS. Although additional experimental techniques will be required to resolve the central nervous system networks that play an instrumental role, it seems reasonable to conclude that alterations in muscle activation dynamics are an expression of at least some of the acute neural adaptations that constitute the basis of cross education.

## Author Contributions

KLR and RGC conception and design of research; KLR, AKR, BK and MK performed experiments; KLR, RGC, AKR, BK and MK analyzed data; KLR, RGC, AD and TJC interpreted results of experiments; KLR prepared figures; KLR and RGC drafted manuscript; KLR, RGC, AD and TJC edited and revised manuscript; KLR, RGC, AD TJC, AKR, BK and MK approved final version of manuscript.

## Funding

UK Biotechnology and Biological Sciences Research Council (ID: BB/I008101/1). This research was also assisted in part by the Department of Education for Northern Ireland. RGC thanks Atlantic Philanthropies for their generous support, through their funding of the NEIL (Neuro-Enhancement for Independent Lives) programme at Trinity College Institute of Neuroscience.

## Conflict of Interest Statement

The authors declare that the research was conducted in the absence of any commercial or financial relationships that could be construed as a potential conflict of interest.

## Abbreviations

ACC: Anterior cingulate cortex
AGLR: Approximated generalized likelihood ratio
AURC: Area under the recruitment curve
CSE: Corticospinal excitability
ECR: Extensor carpi radialis
EMG: Electromyography
FCR: Flexor carpi radialis
IQR: Interquartile range
M1: Primary motor cortex
Mdn: Median
MEP: Motor Evoked Potential
PMd: Dorsal premotor cortex
REML: Restricted maximum liklihood
RMS: Root mean squared
RMT: Resting motor threshold
RoR: Rate of rise
SMA: Supplementary motor area
TMS: Transcranial magnetic stimulation.
